# Profilin-1; a novel regulator of DNA damage response and repair machinery in keratinocytes

**DOI:** 10.1007/s11033-021-06210-6

**Published:** 2021-02-15

**Authors:** Chang-Jin Lee, Min-Ji Yoon, Dong Hyun Kim, Tae Uk Kim, Youn-Jung Kang

**Affiliations:** 1 Department of Biomedical Science, School of Life Science, CHA University, Seongnam-si, Gyunggi-do South Korea; 2 Department of Dermatology, CHA Bundang Medical Center, CHA University, Seongnam-si, Gyunggi-do South Korea; 3 Department of Biochemistry, School of Medicine, CHA University, Seongnam-si, Gyunggi-do South Korea

**Keywords:** Profilin-1, Keratinocyte, Cell integration, DNA damage response, DNA repair

## Abstract

Profilin-1 (PFN1) regulates actin polymerization and cytoskeletal growth. Despite the essential roles of PFN1 in cell integration, its subcellular function in keratinocyte has not been elucidated yet. Here we characterize the specific regulation of PFN1 in DNA damage response and repair machinery. PFN1 depletion accelerated DNA damage-mediated apoptosis exhibiting PTEN loss of function instigated by increased phosphorylated inactivation followed by high levels of AKT activation. PFN1 changed its predominant cytoplasmic localization to the nucleus upon DNA damage and subsequently restored the cytoplasmic compartment during the recovery time. Even though γH2AX was recruited at the sites of DNA double strand breaks in response to DNA damage, PFN1-deficient cells failed to recruit DNA repair factors, whereas control cells exhibited significant increases of these genes. Additionally, PFN1 depletion resulted in disruption of PTEN-AKT cascade upon DNA damage and CHK1-mediated cell cycle arrest was not recovered even after the recovery time exhibiting γH2AX accumulation. This might suggest PFN1 roles in regulating DNA damage response and repair machinery to protect cells from DNA damage. Future studies addressing the crosstalk and regulation of PTEN-related DNA damage sensing and repair pathway choice by PFN1 may further aid to identify new mechanistic insights for various DNA repair disorders.

**Supplementary Information:**

The online version contains supplementary material available at 10.1007/s11033-021-06210-6.

## Introduction

Skin is a primary barrier to detrimental circumstance such as UV and chemical attack. Skin cells, especially keratinocytes in epithelial layer, undergo dynamic changes in response to DNA damage, and a single DNA breakage can be lethal leading to malignant transformation if it remains unrepaired, or aberrantly repaired. Ultra violet radiation B (UVB) is one of the DNA damage agents inducing a wide range of DNA damages, which include not only single-strand DNA breaks (SSBs) and DNA damage adducts such as pyrimidine dimers, but also DNA double strand breaks (DSBs), and each of those products upon DNA damage undergoes suitable DNA repair machinery [1, 2]. DNA DSBs trigger the DNA damage response (DDR), which requires activation of the ataxia-telangiectasia mutated (ATM) kinase–initiated by recruitment of the MRE11, RAD50, and NBS1 (MRN) complex to the sites of DSBs, and subsequent phosphorylation of its downstream substrates involved in various branches of the DDR network that signals cell cycle arrest and DNA repair. Accumulation of DNA damage to both genomic and mitochondria contributes to extrinsic aging-associated skin alterations as well as carcinogenesis. Young individuals with basal cell carcinoma (BCC) have been reported to show poor efficiency of DNA repair in response to extrinsically induced DNA damage compared to healthy groups and exhibit more precocious aging [3]. In addition, previous study has shown that UV-induced DNA damage initiates one of the signals for MMP-1 release, and enhanced DNA repair function reduces MMP-1 expression in human skin cells and tissues [4]. Thus, understanding of mechanisms that underlie the system of DDR and repair is crucial to provide a better therapeutic strategy for skin carcinogenesis and aging.

Profilins are widely known as actin binding proteins that are essential for actin polymerization and cytoskeleton organization [5]. In an actin assembly, profilin catalyzes the exchanges of ADP to ATP of globular actin (G-actin) and it leads to ready state of actin nucleation at barbed end of filamentous actin (F-actin) [6]. Profilins are composed of four isoforms and among these family Profilin1 (PFN1) is the most widely expressed and studied gene emphasizing its roles as a component of actin assembly. Dysregulation of PFN1 induces actin cytoskeleton-associated alterations such as cell proliferation, migration and adhesion with morphological changes [7–9]. However, apart from the roles of PFN1 in cytoskeleton remodeling, it has been reported that PFN1 regulates drug resistance within the interaction of p53 in breast cancer cells and also exhibits the roles in autophagy-mediated drug resistance interacting with the Beclin1 complex in multiple myeloma [10]. In breast cancer, overexpression of PFN1 upregulates PTEN expression following decrease of levels in AKT activation and PFN1-PTEN interaction inhibits IKK phosphorylation leading to suppress NF-kB activation [11]. Despite the extensive research on PFN1, its subcellular functions, especially the functional roles of nuclear compartment of PFN1 in the stressed condition, are yet to be elucidated. Therefore, in this study we firstly characterized the functional roles of PFN1 in human keratinocytes and its specific regulation in DNA damage response and repair machinery.

## Materials and methods

### Cell culture and treatments with DNA damage agents

HaCaT cells kindly gifted from the laboratory of Professor Sangjin Kang from CHA University, are the immortalized human keratinocytes originally derived from the normal skin. HaCaTs were maintained in Dulbecco’s modified Eagle’s medium (DMEM; WELGENE, KOREA) supplemented with 10% fetal bovine serum (FBS; WELGENE, KOREA) and 1% penicillin–streptomycin (WELGENE, KOREA) at 37 °C and 5% CO_2_, and passages between 1 and 10 were used for all experiments. Ultraviolet B (UVB, 20 mJ/cm^2^) radiation using UV Crosslinker (CL-1000 M, UVP, USA) and doxorubicin (DOX, 0.5 μM) were used to induce DNA damage in HaCaT cells. To provide recovery time for cells, cells were washed three times with PBS and maintained in the fresh media supplemented with 10% FBS.

### Cell transfection and infection

The pLKO lentiviral vectors to knockdown PFN1 (shPFN1 construct sequence; CCGGTACGTGAATGGGCTGACACTTCTCGAGAAGTGTCAGCCCATTCACGTATTTTTG) and pCMV-FLAG-PFN1 to overexpress PFN1 were purchased from abcam and Sino Biological, respectively. The corresponding lentiviruses were packaged and generated by transfecting pLKO-lentiviral shPFN1 plasmid or pCMV-FLAG-PFN1 with VSVG and d8.9 into L293 cells. Lentiviral infection was carried out as follows: post transfection with Lipofectamine 2000 (Thermo scientific, USA), viruses were collected after 72 h. After passing through 0.45 μM filters, viruses were used to infect HaCaT cells in the presence of 8 μg/mL polybrene. Following the lentiviral infection, cells were maintained in the presence of puromycin or hygromycin at 2 μg/mL for at least 72 h for selection. Reverse transcription polymerase chain reaction (RT-PCR) and immunoblotting analyses were conducted to validate the levels of PFN1.

### Quantitative real time reverse transcription polymerase chain reaction

SYBR Green (Roche, Basel, Switzerland) assays were used to quantitate DNA damage response and repair pathway related genes in HaCaT cells. Total RNA extracted using TRIzol reagent (Ambion, Life Technologies Corporation, CA, USA) at 1ug was converted to cDNA using M-MLV reverse transcriptase (Promega, Madison, WI, USA), dNTP (Invitrogen, Carlsbad, CA, USA) and oligo dT primer (Labopass, Seoul, Korea). With 1/10 volume of cDNA, gene expression was quantitatively analyzed. Amplifications were run in a CFX Connect™ Real-Time PCR Detection System (BioRad, Hercules, CA, USA). A DNA melting-curve was used to confirm the presence of a single PCR product in each assay. Real-time PCR results for DNA damage response and repair pathway related genes were normalized to β-actin mRNA expression and analyzed using the ordinary one-way ANOVA analysis with Dunnett’s multiple comparison tests. Primer sequence pairs used for these analyses are shown in Supplementary Table 1.

### Immunoblotting analysis

Immunoblotting and subcellular fractionation were performed as previously described [12]. PFN1 (Abcam; ab118984, 1:2000), PTEN (Cell Signaling; 9188, 1:1000), Phospho-PTEN (Ser380/Thr382/383) (Cell Signaling; 9549, 1:1000), AKT (Cell Signaling; 4691, 1:1000), Phospho-Akt (Ser473) (Cell Signaling; 4060, 1:1000), α-Tubulin (Cell Signaling; 2144, 1:2000), SP1 (Santa cruz biotechnology; sc-59X, 1:2000), γH2AX (Abcam; ab11175, 1:2000), Chk1 (Santa cruz biotechnology, sc-377231, 1:1000), Phospho-Chk1 (Ser317) (Cell Signaling; 2344, 1:1000), Chk2 (Santa cruz biotechnology; sc-9064, 1:1000), Phospho-Chk2 (Thr68) (Novus Biologicals; NB100-92502, 1:1000), PARP (Cell Signaling; 9532, 1:1000), Cleaved-PARP(Asp214)(D64E10) (Cell Signaling; 5625, 1:1000) and β-actin (Abcam; ab54724, 1:2000), respectively followed by incubation with IgG-HRP (1:3000, Abcam, UK). Protein bands were visualized by Lumi femto solution (Dogen, Korea). Image J (National Institutes of Health, Bethesda, MD, USA) was used to quantify bands and compare to the loading control.

### Immunofluorescence and microscopy

Immunofluorescence (IF) staining was performed as previous described [12]. HaCaT cells were plated on matrigel-coated cover glasses (1:8 dilution; growth factor-reduced, Corning, USA) in a 24-well plate and grown for 24 h prior to IF staining. Localization studies were performed using antibody to PFN1 (Abcam; ab118984, 1:500), Phalloidin (Abcam; ab176753, 1:1000), Vinculin (Merck; FAK100-Part No.90227, 1:100), E-cadherin (Abcam; ab1416-500, 1:100), EpCAM (Abcam, 187270, 1:100), γH2AX (Abcam; ab11175, 1:300), cleaved caspase 3 (Cell Signaling; 9661s, 1:100), and further incubated with anti-rabbit or anti-mouse IgG fluorescence (Invitrogen). Cover glasses were mounted in Vectashield mountant with DAPI (Vector Laboratories) as nuclear stain. Images were captured using oil immersion 63 × objectives Zeiss 510 microscopy (Carl Zeiss MicroImaging, Röttingen, Germany) and processed using Zen software (ZEISS) and further analyzed with ImageJ (National Institutes of Health, Bethesda, MD) to measure and analyze the intensity of IF staining.

### Wound healing assay

HaCaT cells were seeded in a 6-well plate and cultured to 100% confluence. After 24 h of starvation, a linear scratch was generated using a sterile 1 ml pipette tip and the gap distances of wound closure were measured at serial time points up to 72 h. Image J was used to quantify and the gap distances of wound closure.

### Cell proliferation assay

The capacity of proliferation of HaCaTs was determined directly by counting total cell number manually using hemocytometer. 5 × 10^3^ cells were seeded in 96 well plates and the number of cells were counted at 0 h, 24 h, 48 h, 72 h, and 96 h.

### Cell viability assay

Viability assays of HaCaTs were performed using Cell Viability Assay Kit (#QM1000, BIOMAX, Korea) according to the manufacturer’s instructions. 5 × 10^3^ cell were seeded in 96 well plates and 100ul of DMEM media were added onto each well, then incubated for 24 h, 48 h, 72 h, then 10ul QuantiMAX™ was added at intervals of 24 h incubation time. Reaction of QuantiMax mixture (BIOMAX, Korea) was conducted on the shaker for 90mins, then 450 nm wavelength was detected using a Multiskan™ GO Microplate Spectrophotometer (Thermo scientific, USA). Cell viability values are expressed as percent control and are the means of 3 determinations (*P < 0.05, ***P < 0.005).

### Sphere formation assay

HaCaT cells were plated in a 6-well plate at the density of 5 × 10^5^ cells/well in DMEM supplemented with 10% FBS and 1% penicillin–streptomycin with 90 rpm of shaking. After 7 days of incubation, the sphere size was measured under the inverted microscope with 4 × or 10 × objective.

### Cell cycle assay

The cell cycle stage depending on the status of PFN1 in response to DNA damage was analyzed by using propidium iodide (PI) staining kit (Abcam, UK). Cells were harvested and fixed in 70% ice-cold ethanol overnight, washed two times with PBS, and resuspended in PI/RNase A (Abcam, UK) buffer for 15 min at room temperature. Cells were then examined by flow cytometer (Beckman Coulter, USA) and the data were analyzed using CytExpert software (Beckman Coulter, USA).

### Statistical analysis

Statistical significance was determined by a two tailed Student’s *t*-test. A P-value that was < 0.05 was considered statistically significant (P < 0.05(*), P < 0.01(**), P < 0.001(***) and P < 0.0001(****)).

## Results

### Profilin-1 regulates actin formation in HaCaT cells

**Fig. 1:**
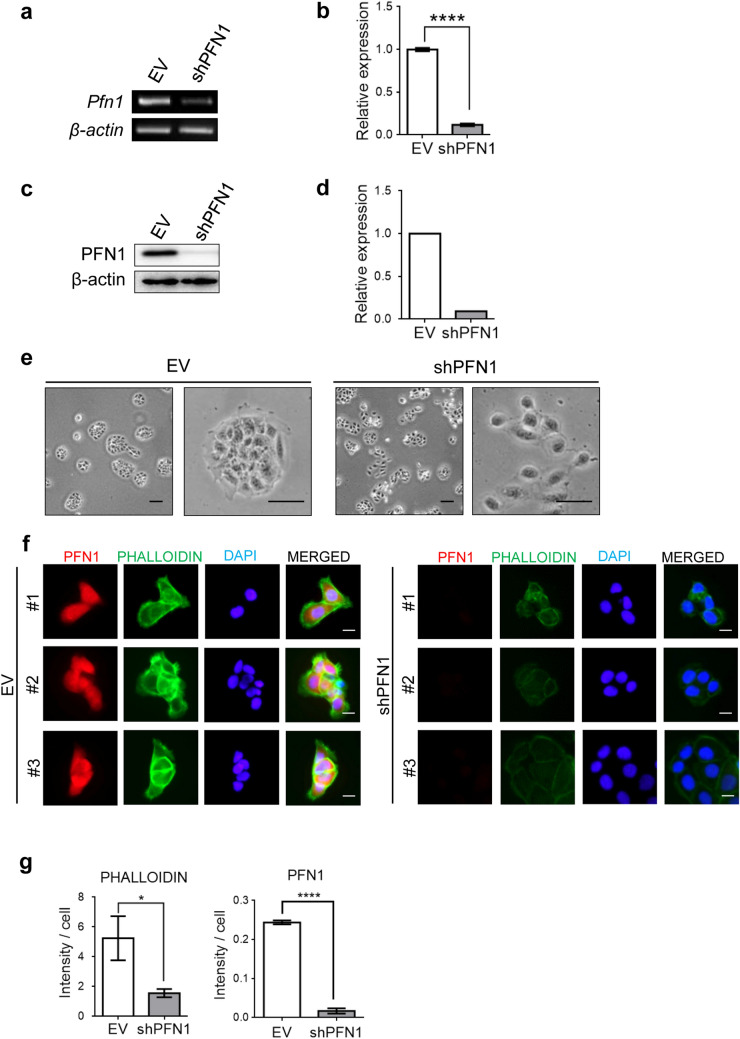
Loss of PFN1 reduces F-actin formation in HaCaT cells. The knockdown efficiency of PFN1 was validated by RT-PCR (**a**), QRT-PCR (**b**), and immunoblotting (**c**, **d**) analyses. **e** Images displaying morphological characteristics of EV- or shPFN1-tranduced HaCaT cells. Scale Bar; 100 um. **f** Co-immunofluorescence (IF) staining of PFN1 and PHALLOIDIN with DAPI nuclear staining in EV- or shPFN1-tranduced HaCaT cells. Scale Bar; 20 um. The relative intensity of PFN1/PHALLOIDIN expression in shPFN1-transduced cells compared to EV cells is summarized in the graphs shown in (**g**). The data represents the means ± SD from three independent experiments. *P < 0.05, ****P < 0.0001

PFN1 plays an important role in actin polymerization by binding to actin and the activity of nucleotide exchange [5, 8, 13]. To examine its roles in human keratinocytes, PFN1 expression was suppressed in HaCaT cells by transducing a lentiviral vector expressing a construct targeting PFN1 shRNA (shPFN1). Depletion of PFN1 was validated by RT-PCR, qPCR and immunoblotting analyses. These analyses showed that shPFN1 transduction in HaCaTs induces approximately tenfold reduction in PFN1 expression at both mRNA and protein levels (Fig. 1a–d). In a culture condition, empty vector(EV)-transduced HaCaTs were grown as a colony whereas PFN1-depleted cells displayed more independent growth (Fig. 1e). In addition, co-Immunofluorescence (co-IF) staining of PFN1 with phalloidin, a marker for filamentous actin examined the effect of PFN1 depletion on actin cytoskeletal reorganization. It revealed that PFN1 depletion induces a significant reduction (− 3.3 fold) of phalloidin expression, particularly weaker at the sites of cell–cell contacts compared to those of control group implicating crucial roles of PFN1 in F-actin formation and cytoskeletal organization in keratinocytes (Fig. 1f, g and Supplementary Fig. 1a).

### PFN1 plays an important role in cell–cell adhesion

**Fig. 2:**
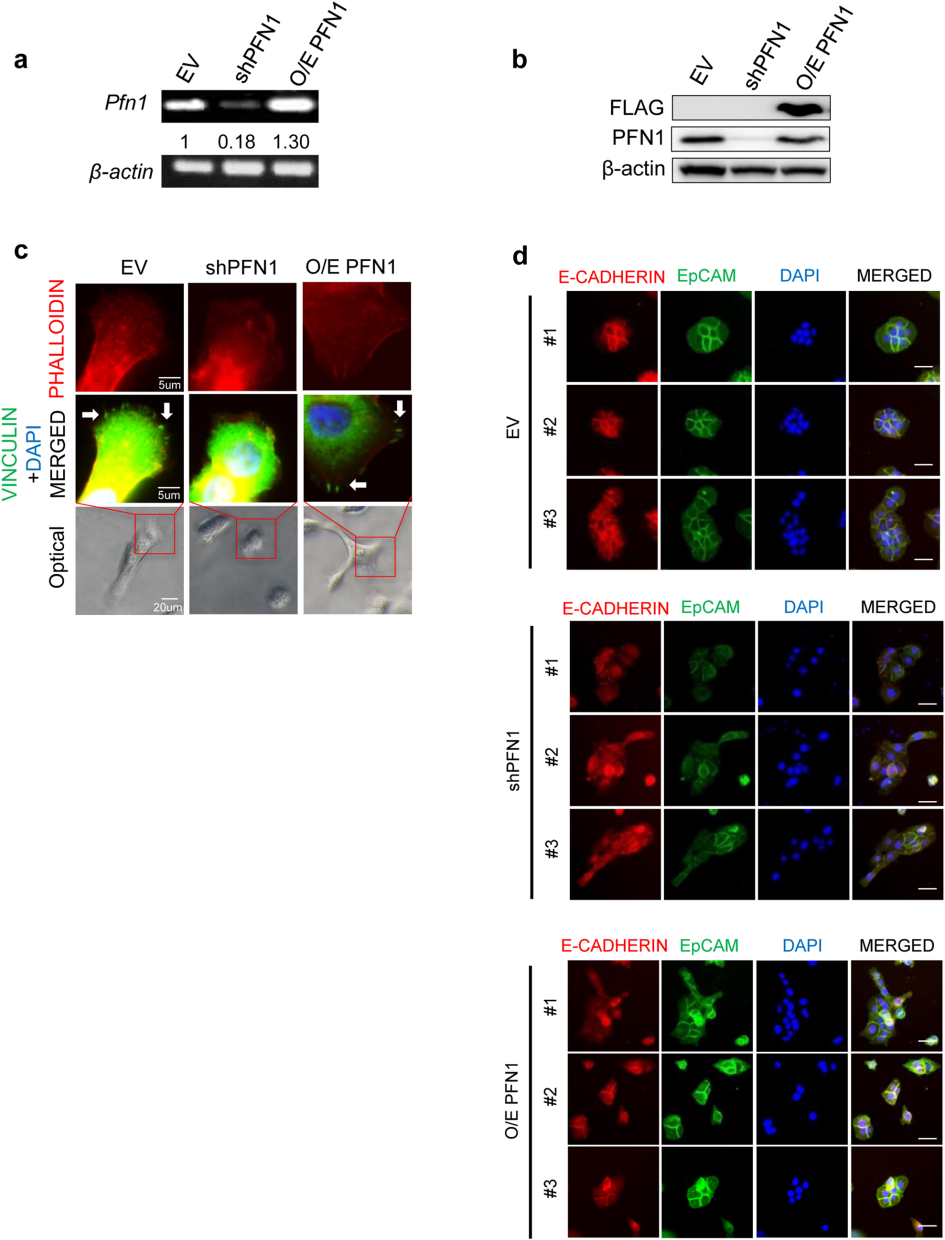
PFN1 participates in cell–cell adhesion. PFN1 knockdown (shPFN1: pLenti-shPFN1 transduction) or overexpression (O/E PFN1: pCMV-FLAG-PFN1 transduction) was validated by RT-PCR (**a**) and immunoblotting (**b**) analyses compared to control (EV: pLenti-EV transduction). β-actin was used for loading control. **c** Co-IF of PHALLOIDIN and VINCULIN with DAPI nuclear staining in EV-, shPFN1, or O/E PFN1-transduced cells. PHALLOIDIN/VINCULIN stained regions are matched to the magnified images from the optical images. **d** Co-IF of E-CADHERIN and EpCAM with DAPI nuclear staining in EV-, shPFN1, or O/E PFN1-transduced cells. Scale Bar; 20 um. Three representative images (#1–#3) of each condition are shown

PFN1 has been recently suggested as a key player in regulating cell–matrix adhesion of human aortic endothelial cells [14, 15]. This led us to ask whether the status of PFN1 has any impact on integrating focal adhesion complex in human keratinocytes. HaCaT cells were transfected with CMV-flag-tagged PFN1 cDNA, which could be distinguished from endogenous PFN1 by anti-flag detection, to induce PFN1 overexpression, and cell–cell adhesion was analyzed in PFN1-overexpressed HaCaTs compared to EV- or shPFN1-transduced cells. The status of PFN1 expression at the condition of knockdown (shPFN1) or overexpression (O/E PFN1) compared to control group (EV) were validated by RT-PCR and immunoblotting analyses (Fig. 2a, b). The impact of PFN1 on cellular adhesive structure at focal adhesion complex was examined by counterstaining of phalloidin with Vinculin in shPFN1-, O/E PFN1, or EV-transduced HaCaT cells. Cells were grown on matrigel-coated cover glass to increase the capacity of cell–cell adhesion. Vinculin is a major focal adhesion plaque protein within a linkage of F-actin and integrins acting as a mechanical clutch to stabilize cell–cell adhesion [16, 17]. Co-IF of Vinculin and phalloidin revealed significant disruption of both F-actin and focal adhesion complex, which is evidenced by reduced expression, especially at the site of cell–cell or cell–matrix contacts. Intracellular localization of Vinculin at focal adhesion was completely disappeared with PFN1 depletion whereas remained highly expressed in control and PFN1-overexpressed cells (Fig. 2c and Supplementary Fig. 2a). Our findings might imply that loss of PFN1 causes Vinculin-dependent integrin-mediated adhesion failure and subsequent attenuation of adhesion-mediated signaling transduction [18]. Interestingly, PFN1-knockdown mediated by shPFN1 transduction induced significant cellular morphological alterations displaying more circular shapes with decreased filopodia protrusions exhibiting low levels of cell–cell adhesion compared to control group (Supplementary Fig. 2b, c). This might imply that loss of actin cytoskeleton organization induced by PFN1 knockdown leads to decrease the ability of cell–cell or cell–matrix adhesion following reduction in filopodia protrusion and morphological alterations. However, little impact was observed in O/E PFN1 group compared to control (Supplementary Fig. 2b, c). Furthermore, intracellular adhesion is regulated by actin cytoskeleton remodeling within a complex of an adherent protein, E-cadherin [15] and actin fibers organize circumferential cables to mediate cell–cell adhesion at the boundaries of cell–cell contacts [19]. Collaborations of actin circumferential cable with adhesion complexes contribute to determine the ability of cell–cell adhesion [20]. IF analyses of phalloidin (Figs. 1b, 2c, and Supplementary Fig. 2a) demonstrated a significant reduction of circumferential actin fiber formation in PFN1-depleted cells compared to control group indicating an important role of PFN1 in actin cytoskeleton organization. To support this notion, co-IF analyses for E-cadherin and EpCAM, widely used cell–cell adhesion markers, were performed. It demonstrated that predominant subcellular localization of both E-cadherin and EpCAM at the sites of cell–cell adhesion was almost disappeared in PFN1-depleted cells whereas both EV- and O/E PFN1-transduced HaCaTs showed clear and obvious boundaries in between of neighboring cells (Fig. 2d). Wound healing assay, which is preferentially related to cell–cell adhesion, revealed that the rates of wound closure were increased with PFN1 knockdown suggesting that a significant reduction in the rates of cell–cell adhesion and cell–cell interaction at the sites of cell junctions (Fig. 2d) might lead cells to be free to migrate (Supplementary Fig. 2d, e). To further investigate whether these alterations have impacts on cell migration and invasion, we next performed assays for cell migration and invasion using trans-wells. PFN1-depleted HaCaTs showed increased rates of migration and invasion than other two groups. However, little differences were observed between PFN1-onverexpressed cells and control (Supplementary Fig. 2f, g). These observations might implicate that PFN1 regulates cytoskeleton organization, which is an important phenotypical standard determined by cellular ability to adhere and transduce signals to adjacent cells.

### PFN1 participates in human keratinocyte proliferation

To verify whether PFN1 plays a role in cell proliferation of human keratinocytes, we analyzed the rates of cell proliferation by measuring cellular dehydrogenase activities at different time points (0–96 h) in the presence or absence of PFN1 in HaCaTs. It has been previously reported that balanced levels of PFN1 is essentially required for the stemness of breast cancer cell, MB-231 [21, 22]. Compared to control group, PFN1-overexpressed HaCaTs showed slower growth rates whereas much accelerated growth was observed in PFN1-depleted cells (Supplementary Fig. 3a). In addition, PFN1-depleted HaCaTs exhibited elevated levels of p-ERK at Thr202/Tyr204 (1.4-fold) compared to EV-transduced cells, while PFN1-overexpressed cells showed dramatically decreased levels of p-ERK (0.6-fold) (Supplementary Fig. 3b, c), which are consistent with previous findings [23]. These results implicate that disruption of actin cytoskeletal organization and cell–cell adhesion induced by PFN1 knockdown has an effect on cell proliferation via ERK-mediated signaling activation. To further investigate roles of PFN1 in anchorage-independent growth, HaCaT cells were plated in a 6-well plate with 90 rpm of shaking, which allows virtually no cell attachment to generate 3-dimensional (3D) culture condition. On day 7 of 3D culture sizes and numbers of individual spheroids were measured. Compared to EV-transduced spheroids, shPFN1-spheroids were much smaller (0.3-fold) and O/E PFN1-transduced spheroids were 3-times larger in size (Supplementary Fig. 3d, e). In 3D environment, cell growth requires collaboration of various cellular features such as cell adhesion, cell–cell interaction and polarity, which are distinct from those of 2-dimensional (2D) culture system [24]. This might be evidenced by our analyses for the impact of PFN1 on cell proliferation based on comparison between anchorage-dependent and anchorage-independent assays. Interestingly, it revealed that PFN1 depletion accelerates cell proliferation in anchorage-dependent assays, however, PFN1-depleted cells showed less spheroid formation with smaller size compared to control in anchorage-independent assays. Moreover, overexpressed PFN1 exerted little impact on the rates of cell proliferation in anchorage-dependent manner, however, they showed larger sphere size formation than control group in 3D culture condition. These results implicate that cytoskeleton remodeling mediated by PFN1 has a different effect on 2D or 3D growth of skin epithelial cells. This might provide an evidence for different growth rates depending on the status of PFN1 expression in different culture conditions.

### Alterations in PFN1 subcellular localization upon DNA damage response and repair

**Fig. 3:**
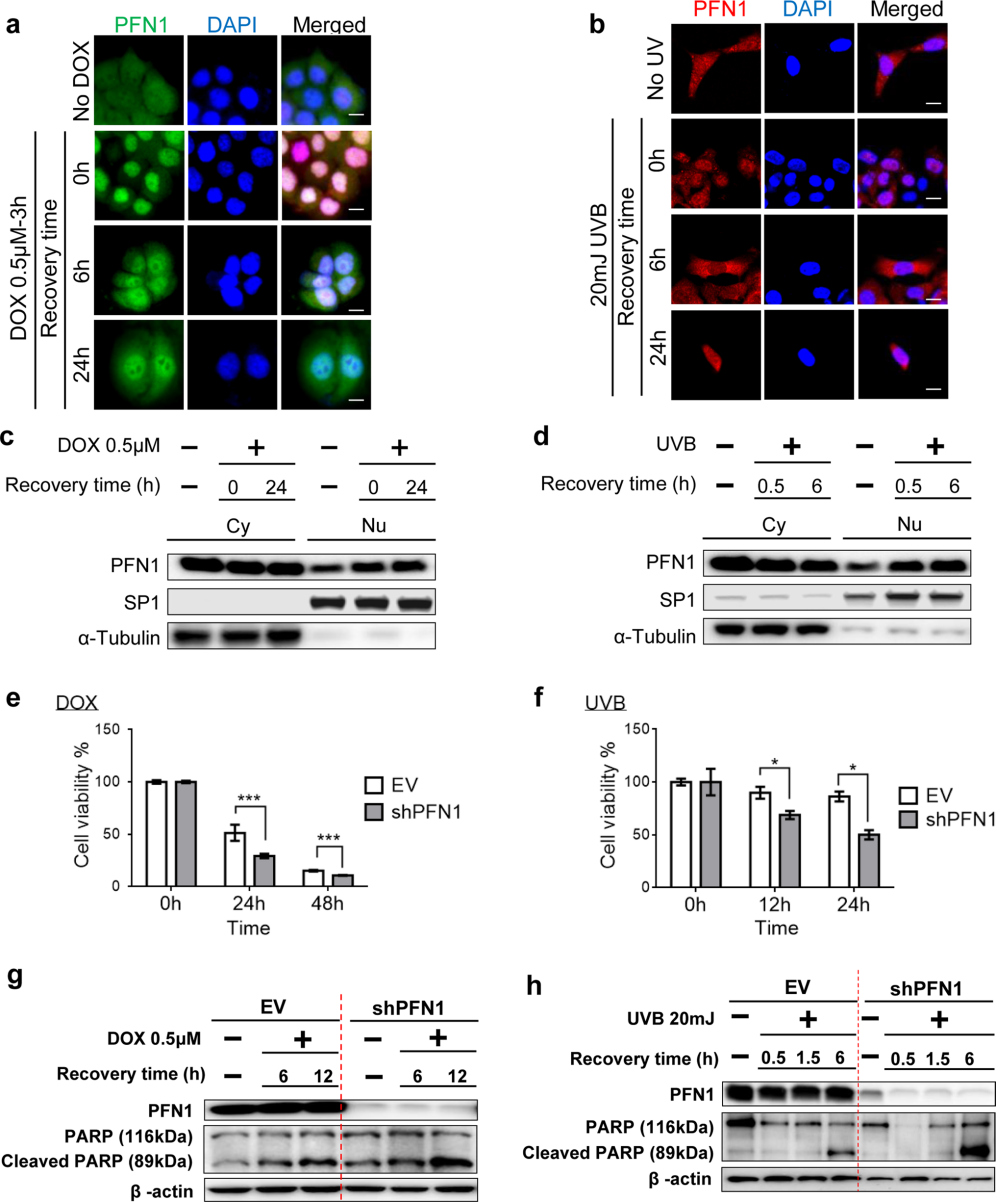
Alteration of subcellular localization of PFN1 upon DNA damage and PFN1 depletion accelerates DNA damage-induced apoptosis. **a** IF of PFN1 with DAPI nuclear staining in the absence of doxorubicin (No DOX) or after 3 h of exposure to DOX (0.5 μΜ) followed by 6 h or 24 h of recovery time in HaCaT cells. Scale Bar; 20 um. **b** Immunoblotting analysis of nuclear (N) and cytosolic (Cy) distributions of total PFN1 in No DOX, or DOX (0.5 μΜ) treatment followed by 24 h of recovery time. Loading controls: SP1 (nuclear) and α–tubulin (cytosolic). **c** IF of PFN1 with DAPI nuclear staining in HaCaT cells with No UVB, or UVB (20 mJ) exposure followed by 6 h or 24 h of recovery time. Scale Bar; 20 um. **d** Immunoblotting analysis of nuclear (N) and cytosolic (Cy) distribution of PFN1 in No UVB, or UVB (20 mJ) exposure followed by 0.5 h or 6 h of recovery time. Loading controls: SP1 (nuclear) and α–tubulin (cytosolic). Cell viability assay of EV- or shPFN1-transduced HaCaT cells in response to doxorubicin (DOX) (**e**) or UVB (**f**) exposure up to 48 h. **g** Immunoblotting analysis of PFN1, PARP, and Cleaved PARP in EV- or shPFN1-transduced HaCaT cells. Cells were exposed to DOX (0.5 μΜ) for 0 h, 3 h, and allowed to recover 6 h or 12 h. β-actin was used for loading control. **h** Immunoblotting analysis of PFN1, PARP, and Cleaved PARP in EV- or shPFN1-transduced HaCaT cells. Cells were exposed to UVB (20 mJ), and allowed to recover for 0.5 h, 1.5 h or 6 h. β-actin was used for loading control

It has been recently reported that the subcellular localization of PFN1 is regulated by exportin 6 and amyotrophic lateral sclerosis(ALS)-related PFN1 mutations have cytoplasmic inclusions and decrease of nuclear import suggesting a critical role of nuclear compartment of PFN1 [25, 26]. Although cytoplasmic PFN1 has been extensively studied as a key modulator of actin polymerization and cytoskeletal reorganization, its nuclear functions are still poorly understood. This led us to examine the subcellular localization of PFN1 in human keratinocytes in the stressed condition with external stimuli inducing DNA damage. Cells were treated with doxorubicin (DOX) or ultraviolet radiation B (UVB) to induce DNA damage and subsequently 6–24 h time was given for cells to recover after DNA damage. Consistent with the previously reported findings [27], PFN1 was predominantly cytoplasmic but still present in the nucleus in the absence of DOX or UVB in culture. Following 0.5 μM of DOX or 20 mJ of UVB exposure, its cytoplasmic localization was significantly changed to the nucleus, and subsequently nuclear localization of PFN1 was restored back to the cytoplasm during the recovery time (Fig. 3a–d, Supplementary Fig. 4a, b). These observations implicate that nuclear PFN1 might be required for responding to the DNA damage and the process of repair function.

### PFN1 depletion accelerates DNA damage-induced apoptosis

We next seek to determine whether PFN1 has roles in regulating DNA damage-induced apoptosis in HaCaT cells. DOX (0.5 μM) or UVB (20 mJ) was applied to 80% confluent HaCaT cells and the rates of cell viability were analyzed using Cell Viability Assay Kit (BIOMAX, Korea), which detects cellular dehydrogenase activity by measuring absorbance (Fig. 3e, f). Interestingly, loss of PFN1 induced no changes in cell viability of keratinocytes in a steady state. However, PFN1 depletion accelerated DNA damage-mediated apoptosis, which was supported by increased levels of cleaved-PARP and cleaved-caspase3, well-known apoptosis markers (Fig. 3g, h, Supplementary Fig. 5a–d), suggesting a crucial role of PFN1 in DNA damage-mediated apoptosis via apoptosis-related PARP signal transduction.

### PFN1 deficiency promotes G1/S arrest and cell apoptosis via PTEN-AKT disruption in response to DNA damage

**Fig. 4:**
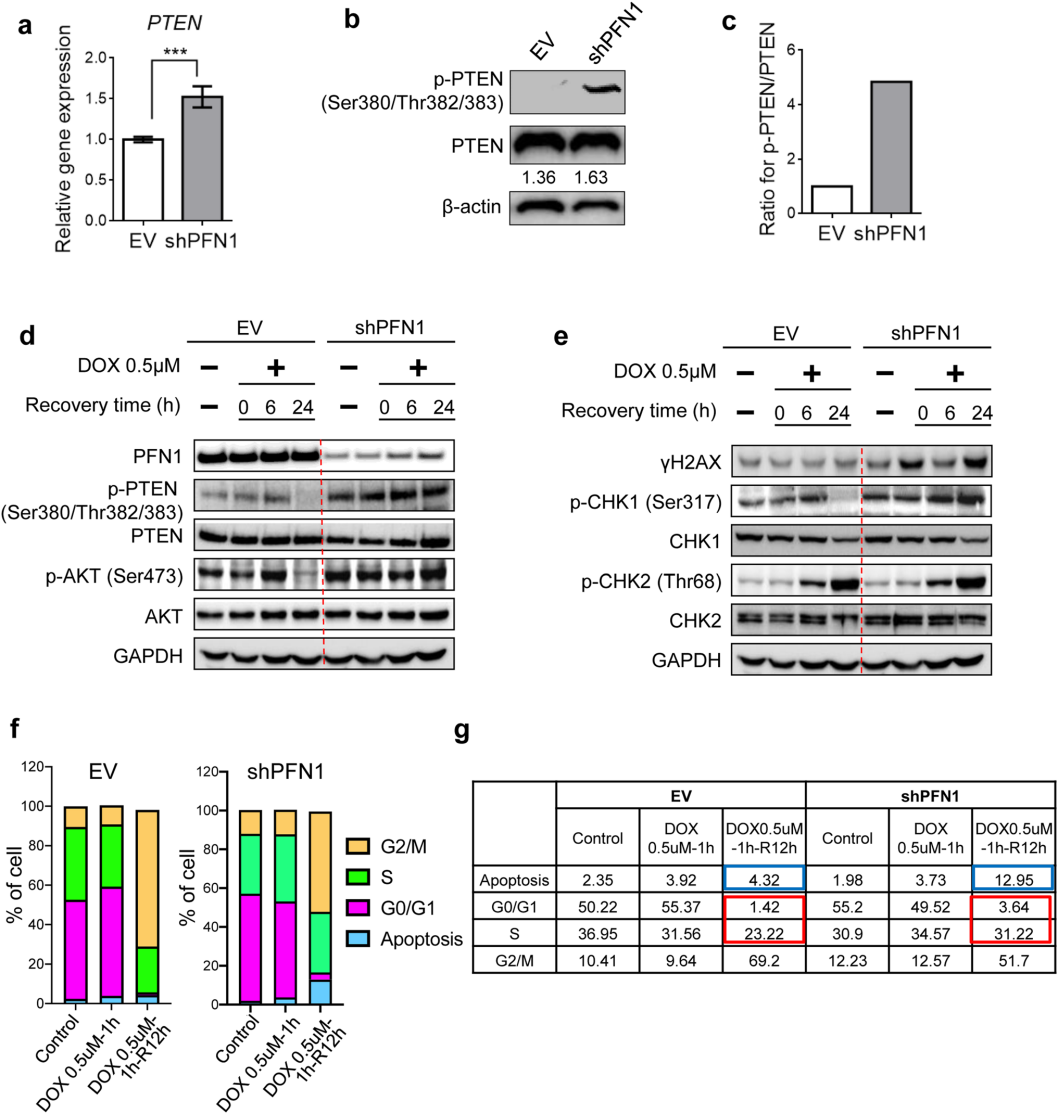
PFN1 has roles in DNA damage signaling in response to doxorubicin treatment. **a** Real-time reverse transcriptase PCR (RT-PCR) was used to quantitate the mRNA expression of *PTEN* in EV- or shPFN1-transduced HaCaT cells. All values were normalized to β-actin. ***P < 0.001. Data are representative of three biological replicates. All graphs depict mean ± s.e.m. **b** Immunoblotting analyses of p-PTEN (Ser380/Thr382/383) and PTEN expression in EV- or shPFN1-transduced HaCaT cells. β-actin was used for loading control. Each number below the bands for PTEN indicate the relative PTEN expression normalized to β-actin. Ratio for p-PTEN/PTEN in EV- or shPFN1-transduced cells was shown in the graph show in (**c**). **d** Immunoblotting analyses of PFN1, p-PTEN (Ser380/Thr382/383), PTEN, p-AKT (Ser473), and AKT in EV- or shPFN1-transduced cells in the absence or presence of DOX (0.5 μΜ) treatment followed by 0 h, 6 h or 24 h of recovery time. GAPDH was used for loading control. **e** Immunoblotting analyses of γH2AX, p-CHK1 (Ser317), CHK1, p-CHK2 (Thr68), and CHK2 in EV- or shPFN1-transduced cells in the absence or presence of DOX (0.5 μΜ) treatment followed by 0 h, 6 h or 24 h of recovery time. GAPDH was used for loading control. **f** Flow cytometry analyses for cell cycle arrest in EV- or shPFN1-transduced cells. Cells were exposed to DOX (0.5 μΜ) for 0 h or 1 h, and allowed to have 12 h to recover. The proportion of cells for apoptosis, G0/G1, S, and G2/M phases were shown in the table shown in (**g**). For the highlighted comparison between EV- and shPFN1-transduced groups at 12 h of recovery time after 1 h of DOX exposure, numbers for apoptosis are boxed in blue and for G0/G1 in red

It has been recently reported that PTEN regulates DDR and repair machinery by associating with DDR and repair factors through cell signal transduction including PI3K-AKT signaling [12, 28]. To address this, we next examined the correlation between PFN1 and PTEN-mediated DDR and repair signaling. PFN1-depleted HaCaT cells exhibited 1.5-fold increase of PTEN expression at mRNA level and remained similar at protein level. However, interestingly 4.5-fold increased phosphorylated-PTEN was observed in PFN1-depleted cells compared to control in a steady state (Fig. 4a–c). This might suggest that PFN1 plays an important role in regulating the functional activity of PTEN by modulating its C-terminal phosphorylation which results in PTEN loss of function. Upon DNA damage induced by DOX treatment the levels of p-AKT (S473) was highly increased following the elevation of p-PTEN (S380/T382/T383) and both p-AKT and p-PTEN levels were returned back to the basal levels after 24 h of recovery time in normal cells whereas PFN1-depleted HaCaTs showed that PTEN was remained highly phosphorylated independent of recovery time after DNA damage induction (Fig. 4d), implicating that PFN1 depletion might desensitize cells to DOX-induced DNA damage. DNA damaged sites are recognized by ATM or ATR complex and these subsequently initiate DNA repair machinery followed by cell cycle arrest that is regulated by checkpoint kinase family; Checkpoint kinase 1 (CHK1) is a serine/threonine-specific protein kinase which regulates cell cycle arrest between G1/S phase, which process is initiated by ATR complex at the sites of SSBs or DSBs [29]. Checkpoint kinase 2 (CHK2) regulates S/G2 phase cell cycle arrest in response to DNA DSBs, which are recognized by ATM-associated complex [30]. Previously, PTEN loss has been reported to inhibit CHK1 to cause DSBs [31]. We demonstrated the disruption of PTEN-AKT cascade with PFN1-depletion in response to DNA damage. Intriguingly, CHK1-mediated cell cycle arrest was not recovered even after the recovery time exhibiting high levels of γH2AX accumulation at 24 h of recovery time after DOX treatment (Fig. 4e). However, CHK2 activation pattern showed no differences between EV- and PFN1-depleted cells (Fig. 4e). Consistent with these data, flow cytometry analyses for cell cycle arrest upon DOX-induced DNA damage depending on the status of PFN1 expression demonstrated that in PFN1-depleted group more cells underwent DOX-induced apoptosis process (blue boxes; 4.32% vs. 12.95%) and also were arrested in G1/S phase at 12 h of recovery time after DOX treatment (red boxes; 34.86% vs. 24.64%) compared to control cells (Fig. 4f, g).

### PFN1 deficiency attenuates DNA damage response and repair machinery

**Fig. 5:**
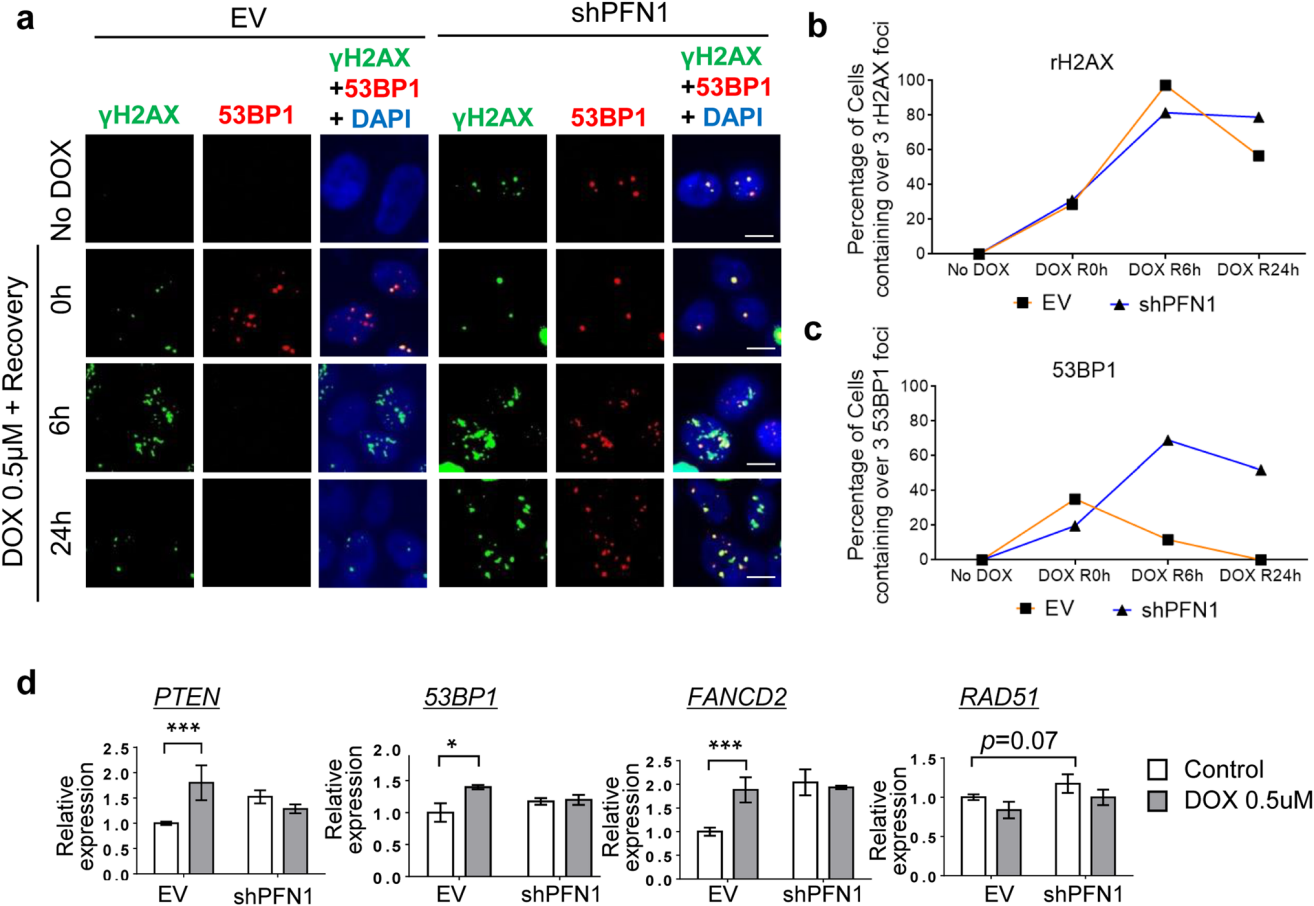
PFN1 deficiency attenuates DNA damage response and repair. **a** IF of γH2AX and 53BP1 with DAPI nuclear staining in EV- or shPFN1-transduced HaCaT cells in the absence or presence of DOX (0.5 μΜ) exposure followed by 0 h, 6 h or 24 h of recovery time. Scale Bar; 20 um. **b** The percentage of γH2AX-positive cell (y-axis) in EV- or shPFN1-transduced cells at the indicated time points of exposure to DOX (No DOX, DOX (0.5 μΜ)) and recovery time (0 h, 6 h, and 24 h). **c** The percentage of 53BP1-positive cell (y-axis) in EV- or shPFN1-transduced cells at the indicated time points of exposure to DOX (No DOX, DOX (0.5 μΜ)) and recovery time (0 h, 6 h, and 24 h). **d** QRT-PCR analyses of *PTEN*, *53BP1*, FANCD2, and *RAD51* in EV- or shPFN1-transduced HaCaT cells in the absence or presence of DOX (0.5 μΜ) exposure. *P < 0.05 ***P < 0.001 ****P < 0.0001. Data are representative of three biological replicates. All graphs depict mean ± s.e.m

To further investigate the status of DNA damage and repair in the presence or absence of PFN1 in response to DNA damage, γH2AX and 53BP1 were co-immunostained and the number of their foci were quantified at each time point after DOX treatment and recovery time. H2A histone family member X (H2AX) is widely used as DNA double strand breakage marker [32] and tumor suppressor p53-binding protein 1 (53BP1) is well-known as a surrogate marker for non-homologous end-joining (NHEJ) DNA repair [33]. Interestingly, cells lacking PFN1 displayed both γH2AX and 53BP1 foci formed in a steady state indicating PFN1 deficiency might induce the accumulation of damaged DNA even in the absence of external damage. Upon DOX-induced DNA damage γH2AX was successfully recruited at the sites of DSBs in both EV- and shPFN1-transduced cells, however damaged cells of PFN1-depleted group failed to recover back to the normal status exhibiting persistent accumulation of γH2AX with higher numbers of foci formed compared to control group (Fig. 5a, b). Furthermore, 53BP1 was recruited and disassociated rapidly in EV-transduced cells upon DOX treatment, whereas PFN1-depleted cells showed slower recruitment upon DOX treatment and 53BP1 foci were remained undissociated even after 24 h of recovery time (Fig. 5a and c). These results led us to investigate the expression levels of four candidate DNA repair-related genes (*PTEN*, *53BP1*, *FANCD2*, and *RAD51*), which might provide a clue for the factors that are essentially affected by PFN1-regulated DDR and repair signaling. Surprisingly, compared to PFN1-depleted group, EV-transduced cells significantly increased the levels of expression of these genes, except for *RAD51*, a surrogate marker for homologous recombination (HR) DNA repair pathway, in the presence of DOX-induced DNA damage (Fig. 5d). Even though further studies will be required to address the detailed mechanisms underlying PFN1-determined DNA repair signaling pathways, these data demonstrate that PFN1 deficiency attenuates DNA damage response and repair function, especially non-HR-related repair system, via aberrantly activated PTEN-AKT signaling pathway and also PFN1 loss induces apoptotic processes by accumulating damaged DNA and arresting cells in G1-S phases of cell cycle (Supplementary Fig. 6). Besides the key roles of PFN1 in cytoskeleton remodeling as an actin-binding protein, our findings strongly suggest the crucial roles of PFN1 as a novel regulator in DNA damage response and repair function within the association of PTEN-AKT axis to protect the cells from extrinsically induced DNA damage.

## Discussion

Cytoskeleton is essential for cell survival, motility and differentiation and its disruption is one of the most distinct features observed in various diseases [34, 35]. Alzheimer’s disease is reported to be related to disruption of neuronal cytoskeleton and also most of cancer is associated with cytoskeleton-mediated morphological changes with aberrant alterations of cellular features including invasion, migration and proliferation [36, 37]. Profilins have four subfamilies and their expression patterns or functions differ in various organs or cell types. For example, although both PFN1 and PFN2 are expressed in breast cancer cells, they have distinct effects on cell migration and invasion ability [38]. In this study, we transduced lentiviral vectors expressing shPFN1 or PFN1 cDNA to manipulate PFN1 expression levels in keratinocyte, HaCaT cells, which exhibit normal morphogenesis and express all the major surface markers of keratinocytes, integrin α6 and CD71 [39–41]. Moreover, upon stimulation HaCaT cells differentiate expressing specific differentiation markers, including Keratins 1/10, filaggrin, and involucrin and also have various inflammatory mediators in response to TNFα or IL-1β, they have been suggested as an appropriate model to investigate the epidermal homeostasis and its pathophysiology, and anti-inflammatory intervention on skin diseases [39, 42, 43]. PFN1 depletion showed dysregulation of F-actin organization such as circumferential actin cables and F-actin stress fibers with morphological changes, strongly suggesting that PFN1 is essential for actin cytoskeleton organization in keratinocytes. Actin cytoskeleton remodeling mediated by PFN1 leads to actin filament-associated functions such as filopodia protrusions and cell adhesion in which actin binding proteins participate in [44]. While PFN1 downregulation decreases the formation of filopodia protrusions and the ability of focal adhesion exhibiting circular morphology, PFN1-overexpressed cells showed filopodia protrusions and focal adhesion at the similar level observed in control group. Furthermore, PFN1-depleted cells showed no F-actin circumferential structures and recruitment of E-cadherin and EpCAM at the surface of cell–cell contacts. Loss of ability of cell–cell adhesion mediated by PFN1 depletion caused significant reduction in sphere formation and also displayed more active migration due to loss of cell–cell adhesion. It failed to recruit Vinculin in PFN1-depleted cells whereas more abundant foci of Vinculin were observed in control and PFN1-overexpressed cells. Based on the results produced from wound healing assay and IF analyses for Vinculin, PFN1 depletion leads cells to be free by losing the ability of cell–cell adhesion, which accelerates wound-closure even though they have lower anchorage ability. Furthermore, while PFN1-depleted cells showed attenuated proliferation with ERK activation in 2D culture condition, PFN1-overexpressed cells displayed slower rates of proliferation with low ERK activation. Interestingly, in 3D culture condition, PFN1 depletion lowers spheroid formation ability instigated by loss of cell–cell adhesion with cytoskeleton disruption, whereas PFN1 overexpression resulted in same or larger sphere size compared to control group. Thus, these results might implicate that molecular mechanisms associated with PFN1 underlying cytoskeleton remodeling in an anchorage-dependent system differ from those of anchorage-independent cell growth condition. It was previously reported that PFN1 deficiency induced suppression of 3D outgrowth with Smad-3 upregulation [45]. In addition, both PFN1 overexpression and downregulation in breast cancer cells decrease the numbers and size of sphere formed in anchorage-independent culture and the loss of PFN1 showed negative effects on tumor-initiating ability with cancer stemness associated transcriptome alteration [46]. These reports indicate that PFN1 participates in determining cellular features of structural remodeling and the regulation of gene expression.

PFN1 is known to have roles in cytoskeleton organization and other cellular features including apoptosis, immune response, stemness, angiogenesis and cell signaling [46–49]. PFN1 is ubiquitously localized to both cytoplasm and nucleus. However, its subcellular function has not been widely studied yet. It has been recently reported that nuclear compartments of actin, which primarily acts within the complex with actin binding proteins like WASP and actin-nucleating ARP2/3, are recruited to damaged chromatin to undergo DNA double strand break repair. Additionally, actin-driven movements induced by polymerization strongly affect double strand break repair, especially by homologous recombination (HR) repair [50]. In our study, we showed alterations of subcellular localization of PFN1 from the cytoplasm to the nucleus upon UVB or doxorubicin-induced DNA damage. Interestingly, PFN1-depleted cells showed decreased sensitivity to DNA damage, which might be occurred through disruption of PTEN-AKT–CHK1 signal cascade. Subcellular fractionation and co-IF analyses demonstrate the lack of nuclear compartment of PFN1 and higher phosphorylated levels of PTEN at C-terminus in PFN1-depleted cells suggesting that nuclear compartment of PFN1 and inactivated nuclear PTEN might be crucial factors that contribute to the attenuation of DDR and repair. These might lead acceleration of DNA damage-induced apoptosis with cell cycle arrest disorder in PFN1-depleted cells displaying more accumulation of γH2AX. Taken together, PFN1 might be suggested as a key factor during the processes of nuclear actin polymerization and DNA damage response and repair, that are strongly correlated together.

In this study, we firstly characterized the functional roles of PFN1 in human keratinocytes and its regulation in DNA damage response and repair machinery. PFN1 depletion induced dysregulation in F-actin organization with disruption of focal adhesion assembly with decreased cell–cell adhesion despite aberrantly proliferative phenotypes with ERK activation in an anchorage-dependent or independent growth. In particular, reduced capacity of cell–cell adhesion led to diminish cell growth in an anchorage-independent condition. PFN1 is ubiquitously observed both in the cytoplasm and the nucleus. However, upon doxorubicin or UVB-mediated DNA damages, the subcellular localization of PFN1 was changed to the nucleus from cytosol, and it returned back to the cytoplasm during recovery time. PFN1 depletion induced PTEN loss of functions instigated by C-terminal phosphorylation. Upon DNA damage PTEN-AKT cascade is preferentially required to be activated to recruit and initiate DNA damage signaling. In the absence of PFN1, DNA damage signal transduction via PTEN-AKT-CHK1 fails to commence and damaged cells are remained unrepaired exhibiting γH2AX accumulation. Future study will extend our findings to other types of cells to confirm the roles of PFN1 as a sensor of DNA damage response and repair signaling. If it is globally applicable, this valuable finding of crosstalk and regulation of DNA damage sensing and repair pathway choice determined by PFN1 may further provide to identify new mechanistic insights for various DNA repair-related disorders.

## Supplementary Information

Below is the link to the electronic supplementary material.

Supplementary file1 (DOCX 14 kb)
Supplementary Table 1. Primer sequence pairs used for RT-PCR analyses.

Supplementary file2 (PPTX 223868 kb)
Supplementary Figure 1. (a) Co-IF of PFN1 and PHALLOIDIN with DAPI nuclear staining in EV- or shPFN1-transduced HaCaT cells. Two representative images are shown. Supplementary Figure 2. (a) Co-IF of PHALLOIDIN and VINCULIN with DAPI nuclear staining in EV-, shPFN1-, or O/E PFN1-transduced HaCaT cells. Two representative images (#1 - #2) are shown. (b) Images of cell morphology in EV-, shPFN1-, or O/E PFN1-transduced HaCaT cells. Circularity of cells from each condition was measured using Image J and quantified in a graph in (c) N.S; not significant, ****P<0.0001. (d) Wound healing assay at 0h, 48h and 96h of time points after linear scratch in EV-, shPFN1-, or O/E PFN1-transduced HaCaT cells. The rates of wound closure were quantified in the graph shown in (e). (f) Cell migration assay of EV-, shPFN1-, or O/E PFN1-transduced HaCaT cells. The rates of migrated cells were quantified in the graph. (g) Cell invasion assay of EV-, shPFN1-, or O/E PFN1-transduced HaCaT cells. The rates of migrated cells were quantified in the graph. Supplementary Figure 3. (a) Cell proliferation assay of shPFN1, or O/E PFN1-transduced cells compared to control HaCaT cells. (b) Immunoblotting analyses of ERK and p-ERK(Thr202/Tyr204) expression in EV-, shPFN1- or O/E PFN1-transduced HaCaT cells. Ratio for p-ERK/ERK (normalized to β-actin loading control) was shown in the graph shown in (c). (d) Spheroid formation assay using shPFN1, or O/E PFN1-transduced cells compared to EV HaCaT cells. (e) The size of spheroids formed in each condition in 6-well round-bottomed plates after 7days in 3D culture was quantified by measuring the spheroid area (μm^2^). Scale bar; 200μm, 500μm. Data represents the means ± SD from duplicate experiments (n=25). ****P<0.0001. Supplementary Figure 4. (a) IF of PFN1 with DAPI nuclear staining in the absence of doxorubicin (No DOX) or after 3h of exposure to DOX (0.5μΜ) followed by 6h or 24h of recovery time in HaCaT cells. (b) IF of PFN1 with DAPI nuclear staining in the absence of UVB (No UVB) or after 3h of exposure to UVB (20mJ) followed by 6h or 24h of recovery time in HaCaT cells. Two representative images are shown. Supplementary Figure 5. (a) IF of cleaved-caspase3 with DAPI nuclear staining in the absence of doxorubicin (No DOX) or after 3h or 5h of exposure to DOX (0.5μΜ) followed by 12h of recovery time in EV- or shPFN1-HaCaT cells. Intensity of cleaved-caspase3 staining (red) was quantified using ImageJ and summarized in a graph shown in (b). (c) IF of cleaved-caspase3 with DAPI nuclear staining in the absence of UVB (No UVB) or after exposure to UVB (20mJ) followed by 3h or 6h of recovery time in EV- or shPFN1-HaCaT cells. Intensity of cleaved-caspase3 staining (red) was quantified using ImageJ and summarized in a graph shown in (d). Supplementary Figure 6. Model summarizes the PFN1 roles in the regulation of DNA damage response and repair machinery. PFN1, which is ubiquitously localized to both cytoplasm and nucleus, regulates actin polymerization and cytoskeletal growth by mediating cell-cell adhesion and filopodia protrusion formation at the sites of cell-cell contact. PFN1 deficiency decreased cell sensitivity to DNA damage, which might be occurred through disruption of PTEN-AKT–CHK1 signal cascade. Upon DNA damage, PFN1 acts as a sensor of DNA damage response and non-HR-related repair signaling, which determines cell fates to survive or die.

## References

[1] Han J, Colditz GA, Samson LD, Hunter DJ (2004). Polymorphisms in DNA double-strand break repair genes and skin cancer risk. Cancer Res.

[2] Koh HK, Geller AC, Miller DR, Grossbart TA, Lew RA (1996). Prevention and early detection strategies for melanoma and skin cancer. Current status. Arch Dermatol.

[3] Wei Q, Matanoski GM, Farmer ER, Hedayati MA, Grossman L (1993). DNA repair and aging in basal cell carcinoma: a molecular epidemiology study. Proc Natl Acad Sci USA.

[4] Dong KK, Damaghi N, Picart SD, Markova NG, Obayashi K, Okano Y, Masaki H, Grether-Beck S, Krutmann J, Smiles KA, Yarosh DB (2008). UV-induced DNA damage initiates release of MMP-1 in human skin. Exp Dermatol.

[5] Alkam D, Feldman EZ, Singh A, Kiaei M (2017). Profilin1 biology and its mutation, actin(g) in disease. Cell Mol Life Sci.

[6] Hurst V, Shimada K, Gasser SM (2019). Nuclear actin and actin-binding proteins in DNA repair. Trends Cell Biol.

[7] Ding Z, Lambrechts A, Parepally M, Roy P (2006). Silencing profilin-1 inhibits endothelial cell proliferation, migration and cord morphogenesis. J Cell Sci.

[8] Witke W (2004). The role of profilin complexes in cell motility and other cellular processes. Trends Cell Biol.

[9] Zou L, Hazan R, Roy P (2009). Profilin-1 overexpression restores adherens junctions in MDA-MB-231 breast cancer cells in R-cadherin-dependent manner. Cell Motil Cytoskel.

[10] Lu Y, Wang Y, Xu H, Shi C, Jin F, Li W (2018). Profilin 1 induces drug resistance through Beclin1 complex-mediated autophagy in multiple myeloma. Cancer Sci.

[11] Zaidi AH, Manna SK (2016). Profilin-PTEN interaction suppresses NF-kappaB activation via inhibition of IKK phosphorylation. Biochem J.

[12] Kang YJ, Balter B, Csizmadia E, Haas B, Sharma H, Bronson R, Yan CT (2017). Contribution of classical end-joining to PTEN inactivation in p53-mediated glioblastoma formation and drug-resistant survival. Nat Commun.

[13] Ding Z, Bae YH, Roy P (2012). Molecular insights on context-specific role of profilin-1 in cell migration. Cell Adh Migr.

[14] Moldovan NI, Milliken EE, Irani K, Chen J, Sohn RH, Finkel T, Goldschmidt-Clermont PJ (1997). Regulation of endothelial cell adhesion by profilin. Curr Biol.

[15] Bachir AI, Horwitz AR, Nelson WJ, Bianchini JM (2017). Actin-based adhesion modules mediate cell interactions with the extracellular matrix and neighboring cells. Cold Spring Harb Perspect Biol.

[16] Calderwood DA, Shattil SJ, Ginsberg MH (2000). Integrins and actin filaments: reciprocal regulation of cell adhesion and signaling. J Biol Chem.

[17] Carisey A, Ballestrem C (2011). Vinculin, an adapter protein in control of cell adhesion signalling. Eur J Cell Biol.

[18] Fukami K, Endo T, Imamura M, Takenawa T (1994). alpha-Actinin and vinculin are PIP2-binding proteins involved in signaling by tyrosine kinase. J Biol Chem.

[19] Vasioukhin V, Fuchs E (2001). Actin dynamics and cell-cell adhesion in epithelia. Curr Opin Cell Biol.

[20] Adams CL, Chen YT, Smith SJ, Nelson WJ (1998). Mechanisms of epithelial cell-cell adhesion and cell compaction revealed by high-resolution tracking of E-cadherin-green fluorescent protein. J Cell Biol.

[21] Yun SP, Ryu JM, Jang MW, Han HJ (2011). Interaction of profilin-1 and F-actin via a beta-arrestin-1/JNK signaling pathway involved in prostaglandin E(2)-induced human mesenchymal stem cells migration and proliferation. J Cell Physiol.

[22] Coumans JV, Gau D, Poljak A, Wasinger V, Roy P, Moens PD (2014). Profilin-1 overexpression in MDA-MB-231 breast cancer cells is associated with alterations in proteomics biomarkers of cell proliferation, survival, and motility as revealed by global proteomics analyses. OMICS.

[23] Gille H, Downward J (1999). Multiple ras effector pathways contribute to G(1) cell cycle progression. J Biol Chem.

[24] Burnette DT, Shao L, Ott C, Pasapera AM, Fischer RS, Baird MA, Der Loughian C, Delanoe-Ayari H, Paszek MJ, Davidson MW, Betzig E, Lippincott-Schwartz J (2014). A contractile and counterbalancing adhesion system controls the 3D shape of crawling cells. J Cell Biol.

[25] Stuven T, Hartmann E, Gorlich D (2003). Exportin 6: a novel nuclear export receptor that is specific for profiling.actin complexes. EMBO J.

[26] Wu CH, Giampetruzzi A, Tran H, Fallini C, Gao FB, Landers JE (2017). A Drosophila model of ALS reveals a partial loss of function of causative human PFN1 mutants. Hum Mol Genet.

[27] Diamond MI, Cai S, Boudreau A, Carey CJ, Lyle N, Pappu RV, Swamidass SJ, Bissell M, Piwnica-Worms H, Shao J (2015). Subcellular localization and Ser-137 phosphorylation regulate tumor-suppressive activity of profilin-1. J Biol Chem.

[28] Ming M, He YY (2012). PTEN in DNA damage repair. Cancer Lett.

[29] Baretic D, Maia de Oliveira T, Niess M, Wan P, Pollard H, Johnson CM, Truman C, McCall E, Fisher D, Williams R, Phillips C (2019). Structural insights into the critical DNA damage sensors DNA-PKcs, ATM and ATR. Prog Biophys Mol Biol.

[30] Berger ND, Stanley FKT, Moore S, Goodarzi AA (2017). ATM-dependent pathways of chromatin remodelling and oxidative DNA damage responses. Philos Trans R Soc Lond B.

[31] Puc J, Parsons R (2005). PTEN loss inhibits CHK1 to cause double stranded-DNA breaks in cells. Cell Cycle.

[32] Paull TT, Rogakou EP, Yamazaki V, Kirchgessner CU, Gellert M, Bonner WM (2000). A critical role for histone H2AX in recruitment of repair factors to nuclear foci after DNA damage. Curr Biol.

[33] Kang YJ, Yan CT (2018). Regulation of DNA repair in the absence of classical non-homologous end joining. DNA Repair (Amst).

[34] French SW, Okanoue T, Swierenga SH, Marceau N (1987). The cytoskeleton of hepatocytes in health and disease. Monogr Pathol.

[35] Seitelberger F, Lassmann H, Bancher C (1991). Cytoskeleton pathology in Alzheimer's disease and related disorders. J Neural Transm Suppl.

[36] Iwazaki R, Watanabe S, Otaka K, Ota K, Ono Y, Sato N (1997). The role of the cytoskeleton in migration and proliferation of a cultured human gastric cancer cell line using a new metastasis model. Cancer Lett.

[37] Price LS, Collard JG (2001). Regulation of the cytoskeleton by Rho-family GTPases: implications for tumour cell invasion. Semin Cancer Biol.

[38] Mouneimne G, Hansen SD, Selfors LM, Petrak L, Hickey MM, Gallegos LL, Simpson KJ, Lim J, Gertler FB, Hartwig JH, Mullins RD, Brugge JS (2012). Differential remodeling of actin cytoskeleton architecture by profilin isoforms leads to distinct effects on cell migration and invasion. Cancer Cell.

[39] Colombo I, Sangiovanni E, Maggio R, Mattozzi C, Zava S, Corbett Y, Fumagalli M, Carlino C, Corsetto PA, Scaccabarozzi D, Calvieri S, Gismondi A, Taramelli D, Dell'Agli M (2017). HaCaT cells as a reliable in vitro differentiation model to dissect the inflammatory/repair response of human keratinocytes. Mediat Inflamm.

[40] Tani H, Morris RJ, Kaur P (2000). Enrichment for murine keratinocyte stem cells based on cell surface phenotype. Proc Natl Acad Sci USA.

[41] Micallef L, Belaubre F, Pinon A, Jayat-Vignoles C, Delage C, Charveron M, Simon A (2009). Effects of extracellular calcium on the growth-differentiation switch in immortalized keratinocyte HaCaT cells compared with normal human keratinocytes. Exp Dermatol.

[42] Jung MH, Jung SM, Shin HS (2016). Co-stimulation of HaCaT keratinization with mechanical stress and air-exposure using a novel 3D culture device. Sci Rep.

[43] Boukamp P, Petrussevska RT, Breitkreutz D, Hornung J, Markham A, Fusenig NE (1988). Normal keratinization in a spontaneously immortalized aneuploid human keratinocyte cell line. J Cell Biol.

[44] Nejedla M, Sadi S, Sulimenko V, de Almeida FN, Blom H, Draber P, Aspenstrom P, Karlsson R (2016). Profilin connects actin assembly with microtubule dynamics. Mol Biol Cell.

[45] Chakraborty S, Jiang C, Gau D, Oddo M, Ding Z, Vollmer L, Joy M, Schiemann W, Stolz DB, Vogt A, Ghosh S, Roy P (2018). Profilin-1 deficiency leads to SMAD3 upregulation and impaired 3D outgrowth of breast cancer cells. Br J Cancer.

[46] Jiang C, Ding Z, Joy M, Chakraborty S, Kim SH, Bottcher R, Condeelis J, Singh S, Roy P (2017). A balanced level of profilin-1 promotes stemness and tumor-initiating potential of breast cancer cells. Cell Cycle.

[47] Romeo GR, Pae M, Eberle D, Lee J, Shoelson SE (2013). Profilin-1 haploinsufficiency protects against obesity-associated glucose intolerance and preserves adipose tissue immune homeostasis. Diabetes.

[48] Yang D, Wang Y, Jiang M, Deng X, Pei Z, Li F, Xia K, Zhu L, Yang T, Chen M (2017). Downregulation of profilin-1 expression attenuates cardiomyocytes hypertrophy and apoptosis induced by advanced glycation end products in H9c2 cells. Biomed Res Int.

[49] Simons M, Schwartz MA (2012). Profilin phosphorylation as a VEGFR effector in angiogenesis. Nat Cell Biol.

[50] Caridi CP, D'Agostino C, Ryu T, Zapotoczny G, Delabaere L, Li X, Khodaverdian VY, Amaral N, Lin E, Rau AR, Chiolo I (2018). Nuclear F-actin and myosins drive relocalization of heterochromatic breaks. Nature.

