# Supplementary material for: Profilin-1; a novel regulator of DNA damage response and repair machinery in keratinocytes
Source: Mol Biol Rep. 2021 Feb 15;48(2):1439–52. doi: 10.1007/s11033-021-06210-6 (PMC7925496; doi:10.1007/s11033-021-06210-6)
Supplement: Supplementary file 1 — Supplementary file1 (DOCX 14 kb) Supplementary Table 1. Primer sequence pairs used for RT-PCR analyses. [file 11033_2021_6210_MOESM1_ESM.docx]

| PFN1 | Forward | TGACAAGACGCTAGTCCTGC |
| --- | --- | --- |
|  | Reverse | GAGGTCAGTACTGGGAACGC |
| 53BP1 | Forward | AGTTTGTGAGCCCCTGTGAG |
|  | Reverse | TCCTGTAGGACCATCTGGCA |
| FANCD2 | Forward | CAGAATGTGACCCTACGCCA |
|  | Reverse | CTCAATGTCCAGCTCTCGGAA |
| PTEN | Forward | AGGGACGAACTGGTGTAATGA |
|  | Reverse | CTGGTCCTTACTTCCCCATAGAA |
| RAD51 | Forward | GGGAATTCTGAAAGCCGCTG |
|  | Reverse | CCTGGCTTACGCTCCACTTC |
| β-actin | Forward | CATGTACGTTGCTATCCAGGC |
|  | Reverse | CTCCTTAATGTCACGCACGAT |

Supplementary Table 1. Primer sequence pairs used for RT-PCR analyses.
